# Correlates of Selected Indices of Physical Fitness And Duration of Incarceration among Inmates in Some Selected Nigeria Prisons

**DOI:** 10.4314/ejhs.v20i1.69430

**Published:** 2010-03

**Authors:** Sulaiman A Olaitan, Hanif Shmaila, Lamina Sikiru, Isa U Lawal

**Affiliations:** 1 Physiotherapy Department, University of Ilorin Teaching Hospital, Ilorin, Kwara State, Nigeria; 2 Physiotherapy Dept., Faculty of Medicine, Bayero University, Kano, Nigeria; 3 Physiokinetics & Biomedical Technology Department, School of Health Technology, Federal University of Technology, Owerri, Nigeria

## Abstract

**Background:**

Incarceration has been associated with reduced physical activity. However, physical inactivity is a major cause of morbidity and mortality. The aim of the present study was therefore, to evaluate the incidence and relationship between the measures of physical fitness and the duration of incarceration in of inmates in Kano-Nigeria prisons.

**Method:**

A cross-sectional study was done to determine the relationship between the measures of physical fitness and the duration of incarceration of inmates in Kano prisons. Subjects' physical fitness level (cardio-respiratory fitness, body mass index and waist circumference) was assessed using standardized protocols. Simple percentage, Pearson moment correlation test and student's t-test were used to analyze variables of interest.

**Result:**

One hundred and sixteen inmates; 108 (93.1%) males and 8 (6.9%) females participated in the study. The study revealed high (93.1%) prevalence of low cardio-respiratory fitness among inmates and significant correlation between the selected indices of physical fitness (cardio-respiratory fitness, body mass index and waist circumference) and duration of incarceration.

**Conclusion:**

The prevalence of low cardio-respiratory fitness was high among inmates and long period of inadequate physical activity may be implicated as causative factor of low physical fitness among inmates in Kano prison. Prison administration and staff should encourage healthy inmate behavior. Provision of adequate facilities to encourage physical activity and sports participation is highly needed.

## Introduction

Prison is an ancient institution, where diverse types of people, who had run foul of the law, some of them possibly innocent live. Modern prison is not just a mechanism for inflicting punishment on the offenders; it is also centre of rehabilitation as well. The main aim of the prison is to rehabilitate the prisoners and develop work efficiency necessary to earn their livelihood and prevent them from reverting to crime again. As a means of rehabilitation the prisoners are employed in different workshed, which is a source of income to the prison (1).

Incarcerated people in the prisons are treated like caged animals in maximum security prisons especially in Australia, England and America, locked into a space not bigger than an average bathroom, with little opportunity for adequate physical activity. They are separated from loved ones, friends and family and surrounded by people who are often mentally unstable, unpredictable, angry, frustrated and sometimes violent (2).

The relationship between physical activity and health is clear. The surgeon general's report on physical activity and health stated that higher levels of regular physical activity are associated with lower death rates (3). Physical inactivity (sedentary behavior) is a major cause of morbidity and mortality compared to those who are physically active (4). Sedentary people have a substantially increased risk of developing diabetes mellitus, heart diseases and a number of other disabling chronic conditions (4, 5, 6, 7, 8). Guyton and Hall (9) reported that multiple studies have now shown that people who maintain appropriate body fitness, using judicious regimes of exercise and weight control have additional benefits of prolonged life.

Imprisoned people usually have a poor health status and an increased risk to suffer chronic debilitating conditions, co-infection with the HIV and hepatitis C virus and/or opiod dependency. However, it has been reported that supervised exercise training can these debilitating condition and improve the overall physical fitness of incarcerated people (10).

The physical fitness of the citizens has been the prime concern of many countries; several researchers in different countries have assessed the fitness of their children, youth and adults (11, 12, 13, 14, 15). However, data on physical fitness status of prisoners in Nigeria seem to be scarce. Therefore, this study was aimed to determine the correlates of physical fitness indices and duration of incarceration among prisoners in Kano, Nigeria.

## Subjects and Methods

This cross-sectional study was carried out to evaluate the correlates of physical fitness level and duration of incarceration of inmates in Kano prisons, Nigeria. All inmates (convicts and those in awaiting trials) in the selected prisons in Kano metropolis, Central Prison and Goron-dutse Kano prison were used as the population. Playgrounds of these prisons were used. Convenient sampling technique was adopted in screening all the inmates after which purposive sampling technique was employed to recruit the prisoners in the selected prisons. One hundred sixteen apparently healthy inmates (108 males and 8 females) out of 505 screened for the study. The study was conducted between 15 May and 15 June, 2008.

**Inclusion criteria:** Only apparently healthy prisoners, who responded positively to healthiness in the Physical Activity Readiness Questionnaire (PAR-Q) (16) and who volunteered to participate in the study, were recruited.

**Exclusion criteria:** Those with medical, surgical and psychiatry conditions such as diabetics, hypertension and other cardiac, renal, respiratory disease were excluded from the study.

The following anthropometric and physiological measurements were measured: stature, body weight, measure of overall obesity (body mass index [BMI]), measure of abdominal obesity (waist circumference [WC]) (17) using standardized protocol. Blood pressure and VO_2_max (measure of cardiorespiratory fitness) were assessed as described by Lamina and Musa (18), Leger & Lambert, (19). Prison inmates were categorized based on their duration of incarceration as short duration (≤ 2months) or long duration (>12months).

Statistical analyses included descriptive statistics (mean, standard deviation and proportions [%]) and inferential statistics. Student's t-test was computed to determine difference between variables of interest. Pearson's correlation test was computed to determine the relationship between physical fitness indices and duration of incarceration of the prisoners. All the statistical analyses were performed on a microcomputer using SPSS for Windows Version 11.0, Chicago; IL, USA. A probability level of 0.05 or less was used to indicate statistical significance.

The study protocol was approved by ethical committee of Bayero University, Kano and the prison authority. A detailed verbal description of the nature and purpose of the study was made to the prisoners. Only on receipt of the written consent from the officials and verbal consent from prisoners were the subjects considered for inclusion in the study.

## Results

Out of the 505 inmates in the selected prisons, only 116 subjects (108 males and 8 females) fulfilled the inclusion criteria. Their mean (±SD) age, SBP (±SD) and DBP (±SD) was 28.5 (± 7.8) years, 146.7 (±19.1) mmHg and 92.7 (±13.2) mmHg, respectively. The prevalence of low cardio-respiratory fitness (CRF) in Kano prisons was high 108 (93.1). Results indicated significant difference in body weight, CRF, BMI, WC, SBP, DBP and VO_2_ max between the two durations (≤ 12months and > 12months) of incarceration at p<0.05. Detailed descriptive statistics (means and standard deviations) and t-value of prisoners' demographic and selected indices of physical fitness by duration of incarceration is presented in the table 1.

**Table 1:** Descriptive analysis of inmates' demographic characteristics and selected indices of physical fitness by duration of incarceration (N=116)

Variables	≤ 12 months (n=89)		> 12 months (n=27)		t-value	p-value
	X	SD	X	SD		
Age (yrs)	28.28	7.69	29.26	8.07	−.572	.568
Weight (kg)	63.42	8.90	67.59	8.32	−2.167	.032*
Height (m)	1.68	0.08	1.70	0.07	−1.269	.207
BMI (kg/m^2^)	21.96	2.83	23.24	2.56	−2.094	.038*
WC (cm)	76.67	3.55	85.85	3.71	−11637	.000*
SBP (mmHg)	145.65	19.50	154.30	18.16	−2.049	.043*
DBP (mmHg)	93.23	13.70	93.62	12.52	−0.133	.891*
VO_2_max (ml/kg/min)	26.49	8.55	20.56	15.94	2.508	.014*
Duration of Incarceration (months)	4.12	3.56	28.41	14.14	14.857	000*

* Significant, P<0.05

Significant correlation between selected indices of physical fitness (CRF [r= −.363], BMI [r= .205] and WC [r= .513]) and duration of incarceration at

**Figure 1:**
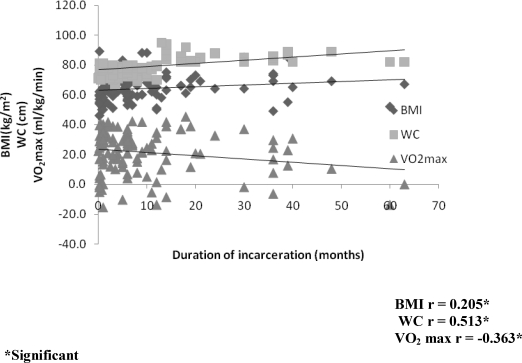
Correlation between duration of incarceration and selected indices of physical fitness.

## Discussion

This study evaluated the prevalence and correlates of indices of physical fitness and the duration of incarceration among prisoners in Kano metropolis. A total of 116 prisoners were recruited in the study out of which 108 were males (93.1%) and 8 were females (6.9%). This is as a result of larger population of male prisoners which is similar to the situation in the United States where the number of incarcerated men far exceeds that of incarcerated women (20). The descriptive analysis outcome of the study showed a high percentage of prisoners with low CRF. However, significant correlation was found between fitness level and duration of incarceration.

Buckaloo et al, (21) in a survey study reported that those who performed aerobic or anaerobic exercise scored significantly lower on the Beck Depression Inventory II and Life Experiences than the inmates who did not exercise. The authors conclude that the lower levels of depression, stress, and anxiety seen among the inmates suggest that exercise is a coping strategy to deal with incarceration. Perez-Moreno et al, (10) studied the effects of a 4-month concurrent cardio-respiratory and resistance training program on the cardio-respiratory fitness, lower and upper body dynamic strength endurance (6-RM test for bench press and knee-extensor exercise, respectively), muscle mass and quality of life (QOL) of adult prison inmates who are HIV/HVC co-infected and enrolled in a methadone maintenance program. They also evaluated a control group. A significant combined effect of group and time was found for peak completed workload (W) (p < 0.01in a gradual cycle ergometer test. A significant combined effect of group and time was also found for both bench press and knee-extensor 6-RM tests, respectively (p < 0.05). They concluded that supervised exercise training can improve the overall physical fitness of incarcerated people.

The correlation between the period of incarceration and the selected indices of physical fitness in the present study is not surprising, incarcerated people have little opportunity for adequate and organized physical activity and exercise particularly in Africa. The correlates between physical inactivity (sedentary or inadequate activity) and cardiovascular and physical fitness has long been established. Studies have shown that supervised exercise can improve the overall physical fitness of incarcerated people. It is well documented that, provided the stimulus is adequate, regular physical activity and endurance exercise training can induce body fat loss and a mobilization of abdominal and visceral adipose tissue (22). In support of this idea (23) recently reported that vigorous physical activity is effective in reducing total and abdominal adiposity. Chronic lifestyle-related diseases are the leading cause of death (24), and leading a sedentary lifestyle is considered a risk factor for the development of many of these diseases as well. Physical activity can produce desirable physiological effects that are protective in nature against these chronic diseases (25). This is true for inmates as well as society.

In conclusion, this study revealed that prevalence of low CRF was high among prisoners and the period of incarceration seems to have an effect on the physical fitness level of prison inmates in Kano-Nigeria. In light of the above described relationships, prison administration and staff should encourage healthy inmate behavior. There is need to provide professionals in prisons who will at intervals educate the inmates on health benefits of physical activity especially exercise and the negative effects of inactivity. Provision of adequate facilities to encourage physical activity and sports participation is highly needed.
